# Radiographic parameters for diagnosing sand colic in horses

**DOI:** 10.1186/1751-0147-50-17

**Published:** 2008-06-13

**Authors:** Anna Kendall, Charles Ley, Agneta Egenvall, Johan Bröjer

**Affiliations:** 1 University Hospital of the Swedish University of Agricultural Sciences, Box 7040, SE-750 07 Uppsala, Sweden; 2 Department of Clinical Sciences, Swedish University of Agricultural Sciences, Uppsala, Sweden

## Abstract

**Background:**

Ingestion of sand can cause colic, diarrhoea and weight loss in horses, but these signs are unspecific and can have many other causes. The amount of sand that induces disease may vary between individuals. To avoid over-diagnosing, it is important to determine the amount of sand that can be found in horses without clinical signs of gastrointestinal disease. The aim of this study was to use previously suggested parameters for establishing a radiographic diagnosis of sand colic, and compare these findings between a sand colic group and a control group.

**Methods:**

Abdominal radiographs were obtained in 30 horses with a complaint unrelated to the gastrointestinal tract. In addition, archived abdominal radiographs of 37 clinical cases diagnosed with sand impaction were investigated. The size of the mineral opacity indicative of sand in the abdomen was measured and graded according to a previously published protocol based on height and length. Location, homogeneity, opacity and number of sand accumulations were also recorded.

**Results:**

Twenty out of 30 control horses (66%) had one or more sand accumulations. In the present study; height, length and homogeneity of the accumulations were useful parameters for establishing a diagnosis of sand colic. Radiographically defined intestinal sand accumulation grades of up to 2 was a common finding in horses with no clinical signs from the gastrointestinal tract whereas most of the clinical cases had much larger grades, indicating larger sand accumulations.

**Conclusion:**

Further work to establish a reliable grading system for intestinal sand content is warranted, but a previously proposed grading system based on measurements of height and length may be an alternative for easy assessment of sand accumulations in the meantime. The present study indicates that a grade 1 – 2 sand accumulation in the intestine is a frequent finding in horses. When working up a case with clinical signs from the gastrointestinal tract, one or more accumulations of this grade should not be considered the cause until other possibilities have been ruled out.

## Background

Ingestion of sand can cause signs of acute or recurrent colic/diarrhoea, weight loss and poor performance [1-5]. Due to the nonspecificity of these changes it is difficult to make a diagnosis merely on the basis of clinical signs. To assist in detecting the presence of intestinal sand, abdominal auscultation, palpation of sand filled viscus per rectum, fecal sand sedimentation test, abdominal ultrasound and abdominal radiography can be used [4-7]. As horses naturally graze, sand can be present in small amounts even in well-managed individuals. Sand excretion in healthy horses has been studied by fecal sand sedimentation test [8] but the method does not provide a quantification of the intestinal sand content and has a low sensitivity as a test for intestinal sand accumulation with a large number of false negatives [1,4]. Due to this it should not be used as a gold standard for diagnosing sand impactions or other sand-related problems. While auscultation of the abdomen has been described as a valuable diagnostic tool to detect sand within the intestine [6], it cannot be used to quantify the sand content. Ultrasound has been evaluated as an aid in diagnosing sand impactions but was less reliable than radiography [5]. Radiographic examination of the abdomen is currently the most useful tool for diagnosing the presence of sand within the intestines, as it is readily performed and can be used quantitatively. Moreover, radiography can also be used to monitor the effects of medical treatment in removing sand from the large colon [4,5,7]. However, the amount of sand required to induce a clinical problem is not known and may vary between individuals [1,3,6]. While as little as 8 kg of sand has been found in horses requiring surgical intervention for sand/gravel impaction [3], horses have been administered up to 10 kg of sand in clinical trials without showing any signs of colic or diarrhoea [6]. Keppie *et al*. recently suggested a scoring system for differentiating clinically significant sand accumulations from accumulations that do not cause colic [7]. The aim of the present study was to use the parameters previously suggested by Keppie *et al. *for establishing a diagnosis of sand colic by the aid of radiography (height, length, homogeneity, opacity and location of mineral opacity), and compare these findings between a sand colic group and a control group. The aim was also to use a previously suggested but not clinically evaluated grading system by Korolainen and Ruohoniemi based on height and length of the mineral opacity suggestive of sand accumulation [5].

## Methods

The study was approved by the Ethical Committee for Animal Experiments, Uppsala, Sweden.

### Horses

#### Control group

The control group contained 30 horses aged 3–22 years (14 Swedish Warmbloods, 6 Standardbreds, 3 Icelandic horses, 2 Thoroughbreds, 1 Holstein, 1 Friesian, 1 Connemara, 1 New Forest and 1 Fjord Horse) presented for medical evaluation of lameness (27), cervical spine compression (1), sinusitis (1) or acute laceration (1). Except for three racehorses (1 Thoroughbred and 2 Standardbreds) the horses were used for pleasure riding at various levels. The control horses were all admitted to the University Animal Hospital of the Swedish University of Agricultural Sciences between March and December 2006, as well as during April 2007. After consent, the owner was asked to fill out a questionnaire regarding clinical history and turn-out. All horses had been turned out daily until the day prior to admission. All but three horses were fed with either hay or silage from the ground or had access to pasture during turn-out. The ground where horses were turned out varied from lush grass pastures to sandy or stony paddocks. Only two horses lacked access to fresh water during turn-out. None of the horses had a history of colic, diarrhoea, anorexia or weight loss during the six months prior to presentation. In order to rule out obvious findings that would indicate problems from the GI-tract, horses were examined prior to inclusion. No sand sounds were audible on abdominal auscultation and incisors were normal on visual examination. Blood was sampled for complete blood cell count and plasma fibrinogen analysis. All horses had clinical and blood parameters within normal limits.

#### Sand impaction group

Clinical cases diagnosed with sand impaction were obtained by searching records from 2005–2007 from three of the largest equine referral practices in Sweden. Two of the clinics have a low incidence of sand impaction (Strömsholm Regional Equine Hospital, Karin Anlén, personal communication and the University Animal Hospital of the Swedish University of Agricultural Sciences) and one clinic has a high incidence (the Regional Animal Hospital of Helsingborg, Anna Johansson, personal communication). In the sand impaction group only horses > 1 year of age diagnosed by the aid of computed radiography of the abdomen were included. Six cases from the University and two cases from Strömsholm met these criteria. From Helsingborg only the first 30 hits from the electronic search of medical records were included. One of these cases was subsequently removed from the analysis because the sand detected radiographically had poorly defined margins that obviated assigning a grading score. This led to a total number of 37 horses (age 3–27 years) in the sand impaction group.

#### Radiography

Radiographs at the radiology department of the Swedish University of Agricultural Sciences were taken with an x-ray tube mounted on an overhead gantry using Fuji digital image plates (Fuji Photo Film Co. Ltd., Japan), processed with a computed radiography system (Fujifilm FCR XG-1, Fuji Medical Imaging Co. Ltd., Japan) and viewed on a dedicated workstation with a picture archiving communication system (PACS, Centricity RA 600 V6.1 Diagnostic, GE Medical Systems, Slough, UK). Image plate cassettes were placed in a ceiling mounted holder. Standing left to right lateral projections of the abdomen were taken using a focal spot film distance of 200 cm. Exposure settings were 150 kVp/100 mAs (horses < 500 kg) or 150 kVp/200 mAs (horses > 500 kg). All horses were sedated with i.v. detomidine (Domosedan, Orion Pharma AB Animal Health, Sollentuna, Sweden) as needed. For the first 20 control horses five projections were taken to include the entire abdomen. Because sand was only found in the most ventral regions, only three projections of the ventral portions of the abdomen were taken for the following 10 control horses in order to avoid unnecessary radiation. To avoid misdiagnosis due to underexposure the ribs on both sides had to be visible in the radiograph of the cranioventral abdomen [9]. In the sand impaction group the number of projections varied, but commonly only two to three projections of the ventral portion of the abdomen were obtained.

Radiographs at Strömsholm Regional Equine Hospital were taken with an x-ray tube mounted on an overhead gantry using Agfa digital image plates (Agfa, Gevaert, Belgium). Standing left to right lateral projections of the abdomen were taken using a focal spot film distance of 180 cm. Radiographs at the Regional Animal Hospital of Helsingborg were taken with an x-ray tube mounted on an overhead gantry using Kodak digital image plates (Carestream Health, Inc. Rochester, N.Y.). Standing left to right lateral projections of the abdomen were taken using a focal spot film distance of 180 cm. Exposure settings at Strömsholm and Helsingborg were not recorded. Radiographs were viewed and evaluated using the PACS at the Swedish University of Agricultural Sciences.

### Measurements

The measurements of sand accumulations were made with the tools available in the PACS, and these measurements were performed by one person (AK). The maximum length and width of the largest sand accumulation was recorded. For curved accumulations the maximum length was measured as a straight line between the ends and not through the curve of the accumulation. If an accumulation was too large to be completely included within one projection, the maximum length within the radiograph was obtained. The following parameters were also recorded: location (specified as cranioventral or other), number of accumulations, opacity compared with a ventral part of a rib on the same image (specified as much less opaque, mix or as opaque as/more opaque than a ventral rib) and homogeneity (specified as heterogeneous, mix or homogeneous).

The sand accumulations were graded on a 0 – 4 scale according to a modification of the scoring system by Korolainen and Ruohoniemi (2002): 0: No sand, 1: < 5 × 5 cm, 2: ≤ 5 × 15 cm or ≤ 15 × 5 cm, 3: ≤ 5 × 15 cm or ≤ 15 × 5 cm close to the ventral abdominal wall, 4: > 5 × 15 cm or >15 × 5 cm. If an accumulation was thin (<5 cm) but longer than 15 cm it was graded as a 4.

### Statistical analysis

Descriptive statistics were calculated with respect to whether the horses were sand colic cases or controls. The Mann-Whitney test was used to compare the number of accumulations, height and length of the accumulations in the sand colic cases and controls. Horses with no accumulations were removed from comparison. The Chi-square test was used for the opacity (equal density versus increased density), homogeneity (homogenous versus mixture) and location (cranioventral versus other). Differences in grade of sand accumulation were analysed using the Mann-Whitney test. First, a comparison between all the control horses (n = 30) and the horses with sand colic (n = 37) was performed. In a second comparison the ten grade 0 horses (no visible sand) were excluded from the control group (n = 20). Data is presented as median (range) and the p-value limit was set to 0.05. The statistical software SAS (SAS Institute Inc., Cary, NC, USA) was used for data handling.

## Results

Of the 30 control horses, 20 (66%) had one or more mineral opacities visible in the radiographs. In the sand colic cases the number of accumulations had a median of 1 (1 – 5) and in the controls the median was 1.5 (1 – 4). There was no statistical difference in number of accumulations between colic and control groups (p = 0.840). The medians for maximal length and height were 265 mm (73 – 400) and 90 mm (12 – 200) in the sand colic cases and 83 mm (7 – 156) and 9 mm (2 – 86) in the controls. The sand colic cases had significantly longer and higher accumulations than the controls (p < 0.001). The results for opacity, homogeneity and location are shown in table 1.

**Table 1:** Distribution of opacity, homogeneity and location of sand accumulations (parameters from Keppie *et al.*) in sand colic cases and controls

		Controls (n = 20)		Sand colic (n = 37)		Chisq
Variable	Categories	Number	Percent	Number	Percent	p-value

Opacity	Equally dense	8	40	3	8	0.004
	More dense	12	60	34	92	
Homogeneity	Homogenous	8	40	30	81	0.002
	Mixture	12	60	7	19	
Location	Cranioventral	25	89	34	92	0.418
	Other	3	11	3	8	

**Figure 1:**
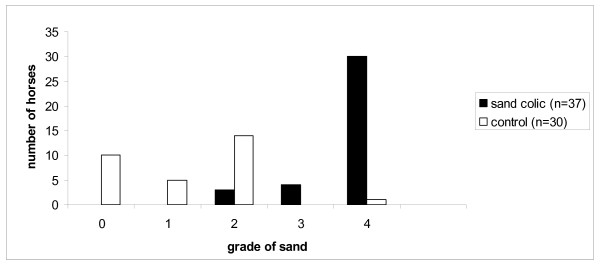
Sand accumulation grades according to Korolainen and Ruohoniemi in sand colic and control groups.

The sand accumulation grades are shown in Figure 1. The median grade of sand accumulation in the control group was 1.5 (range 0 – 4) with all horses included, and 2 (range 1 – 4) when horses with no visible sand were excluded. The median grade for the sand impaction cases was 4 (range 2 – 4). There was a statistical difference in sand accumulation grade between the two groups which remained regardless of whether the grade 0 control horses (no visible sand) were included or excluded from comparison (p < 0.001).

## Discussion

Despite the fact that the study of the control horses was carried out in an area of low incidence of sand impaction, 66% of these horses had one or more sand accumulations within the intestine. It has previously been shown that healthy horses excrete sand in the feces [8] and a reference value is therefore needed in order to diagnose abnormal amounts of intestinal sand. When using the modified grading system by Korolainen and Ruohoniemi [5], a sand accumulation of grade 1 or 2 was a common finding in the control group, whereas most of the clinical cases with sand impaction had much larger accumulations, as reflected in the higher grade. There was a small overlap in grades of sand between the two groups of horses. The control horse that was graded as a 4 had a rounded accumulation of 9 × 11 cm. Even though the accumulation was fairly small it did not fit into the lower grades. This is a weakness of the scoring system as elongated and thin or very rounded sand accumulations are probably weighted excessively in the grading scheme. The radiographs of 10 control horses did not cover the entire abdomen. This may have caused a biased grading of the control horses if sand accumulations were missed. However, this is not likely since all the mineral opacities in the first 20 control horses were found in the cranioventral part of the abdomen. In all horses with sand accumulation, the sand was visible in one of the two projections of the most cranioventral abdomen. These views coincide with the anatomical location of the large colon.

The scoring system by Keppie *et al. *[7] was proven to have good repeatability and was more reliable than subjective assessment of mineral opacities. In addition to measuring the size of the accumulation, parameters such as opacity, location, homogeneity and number of accumulations were in that study found to be significantly different between sand colic cases and controls. When applying the parameters included in the scoring system by Keppie *et al. *to our cases, location of the sand impaction and number of accumulations were not statistically different between groups. In the present study there was a statistical difference between groups when opacity and homogeneity were evaluated, but when opacity was compared according to the scoring system by Keppie *et al*. (accumulations more opaque than or as opaque as a rib were graded similarly), no difference was found. This left homogeneity, height and length as valuable parameters for separating sand colic cases from controls. There are several reasons for discrepancies between this study and the previously published study [7]. The control cases in the present study were not presented for problems related to the gastrointestinal tract. In the previous study controls were selected on the basis of abdominal radiography without a subsequent diagnosis of sand colic. In the previous study, more than 30% of the cases included were foals, whereas only mature horses (≥ 3 years) were included in the present study. Foals have an immature gastrointestinal tract and may not be adequate for comparison with mature horses.

In the study by Keppie *et al*. [7], height and length of the accumulation was standardised to the width of the mid-body of a rib. This method accounts for the size of the horse as smaller horses are likely to tolerate smaller amounts of sand than larger individuals. It also decreases the effect of magnification in the radiograph. However, in most cases in the present study it was not possible to measure the width of the mid-portion of the rib in the radiograph of the cranioventral abdomen (where almost all of the accumulations were located). Despite the use of computed radiography, there was poor contrast between the mid-body of the rib and the surrounding soft tissue opacity (thin mineral opacity vs. thick soft tissue opacity) which resulted in the margins of the rib being poorly defined and making some measurements uncertain. Using the measurements from a more dorsal position on the rib in another image could lead to significant magnification errors, since the distance between the horse and the cassette may have changed between images. It would have been interesting to assign grades according to the scoring system by Keppie *et al. *to the horses in the present study, but this was not possible due to the problems with measuring the ribs. A metal clip with a length standardized to the size of the horse could have been taped to the abdomen prior to exposure to overcome part of this problem. However, the abdomen of a 500 kg horse can have a width of more than 50 cm. If the accumulation is located in a part of the abdomen closest to the x-ray tube, the size of the accumulation relative to a rib on the side closest to the film will be overestimated. To evaluate this, bilateral images would have to be obtained, or bilateral clips could be placed on the abdomen and a mean value of the length could be used. Using linear markers may however also be a problem as the length will be underestimated if they are not placed perpendicular to the x-ray beam. Computed radiography is becoming widely used and allows for post-processing of images such as compensation for underexposure which is the major cause of misdiagnosis [10] when looking for sand. A disadvantage of radiographic evaluation of intestinal sand content is that it provides a 2-dimensional measurement of a 3-dimensional structure. Also, the location of sand within the abdomen (and hence the amount of magnification) can not be easily established.

Horses consume sand when it is mixed with hay fed on the ground, when they graze and when drinking from shallow muddy pools [11,12]. Some horses will deliberately eat sand for unknown reasons [12]. Sand colic often occurs in a single individual within a herd, which raises the question of whether there has to be a predisposition to the accumulation of sand such as decreased intestinal motility. On the other hand, sand accumulations could potentially cause inflammation of the intestinal mucosa [11] with disrupted motility patterns and decreased excretion as a result. Seasonal variation in intake with less access to sand in the winter could have led to a lower mean grade of sand in the control group and an underestimation of normal amounts of sand within the intestines. Therefore, no control horses were radiographed between January and March 2007 as the ground was frozen and/or covered with snow during these months.

The control and sand colic groups in this study are not completely comparable, as the radiographs of the clinical cases were obtained in different clinics and from different areas with varying incidence of sand colic. In this study, one of the three equine practices (Helsingborg) had more cases of sand impaction in one year than the other two clinics had in three years. A between-practice comparison of insured horses with colic in the statistics from Agria Insurance between 1997 and 2004 also showed marked differences in frequency of sand impactions (data not shown). Combining veterinary care and life-insurance claims and calculating on the basis of receipts, less than 1% of the colics were diagnosed as sand related in the two clinics located in the central Sweden (the University Clinic and Strömsholm), although in Helsingborg located in the southern part of the country, 6% were diagnosed as caused by sand [13]. These numbers could be biased by different routines and clinicians, but are supported by the results of this study and by previous observations. Regional variation in incidence of sand-related colic has been reported anecdotally by several authors [12,14,15]. The reason for the observed differences could be variable access to thawed ground or different types of soil and pasture. However, these factors were not recorded in the present study.

## Conclusion

Grading of parameters such as opacity of the mineralisation may help to differentiate normal sand accumulations from clinically significant; however some of the parameters previously proposed by Keppie *et al. *[7] were not found to be valuable diagnostic tools when evaluated in the present study. Further work to establish a reliable grading system for intestinal sand content is warranted, and the grading system proposed by Korolainen and Ruohoniemi [5], based on measurements of height and length, may be an alternative for easy assessment of sand accumulations in the meantime. The present study indicates that a grade 1–2 sand accumulation in the intestine is a frequent finding in horses. When working up a case with clinical signs from the gastrointestinal tract, one or more accumulations of this grade should not be considered the cause until other possibilities have been ruled out.

## Authors' contributions

AK, CL and JB participated in the design of the study, AK conceived of the study, performed the measurements and drafted the manuscript, but all authors have contributed substantially to the final manuscript. AE performed the statistical analysis in cooperation with JB. All authors read and approved the final manuscript.
